# Supplementary material for: Understanding resonant charge transport through weakly coupled single-molecule junctions
Source: Nat Commun. 2019 Oct 11;10:4628. doi: 10.1038/s41467-019-12625-4 (PMC6789103; doi:10.1038/s41467-019-12625-4)
Supplement: Supplementary file 1 — Supplementary Information [file 41467_2019_12625_MOESM1_ESM.pdf]

Supplementary Information:
Understanding resonant charge transport through weakly coupled single-molecule junctions

Thomas et al.

Contents

**Supplementary Note 1**	**Chemical structures**	**4**
**Supplementary Note 2**	**Theory**	**6**
**Supplementary Note 3**	**DFT calculations**	**10**
**Supplementary Note 4**	**Substrate reorganisation energy**	**19**
**Supplementary Note 5**	**Supporting data for device A**	**21**
**Supplementary Note 6**	**Supporting 5 K data (device E)**	**23**
**Supplementary Note 7**	**Supporting 77 K data**	**24**
**Supplementary Note 8**	**Alternative intermediate-temperature models**	**29**
**Supplementary References**		**32**

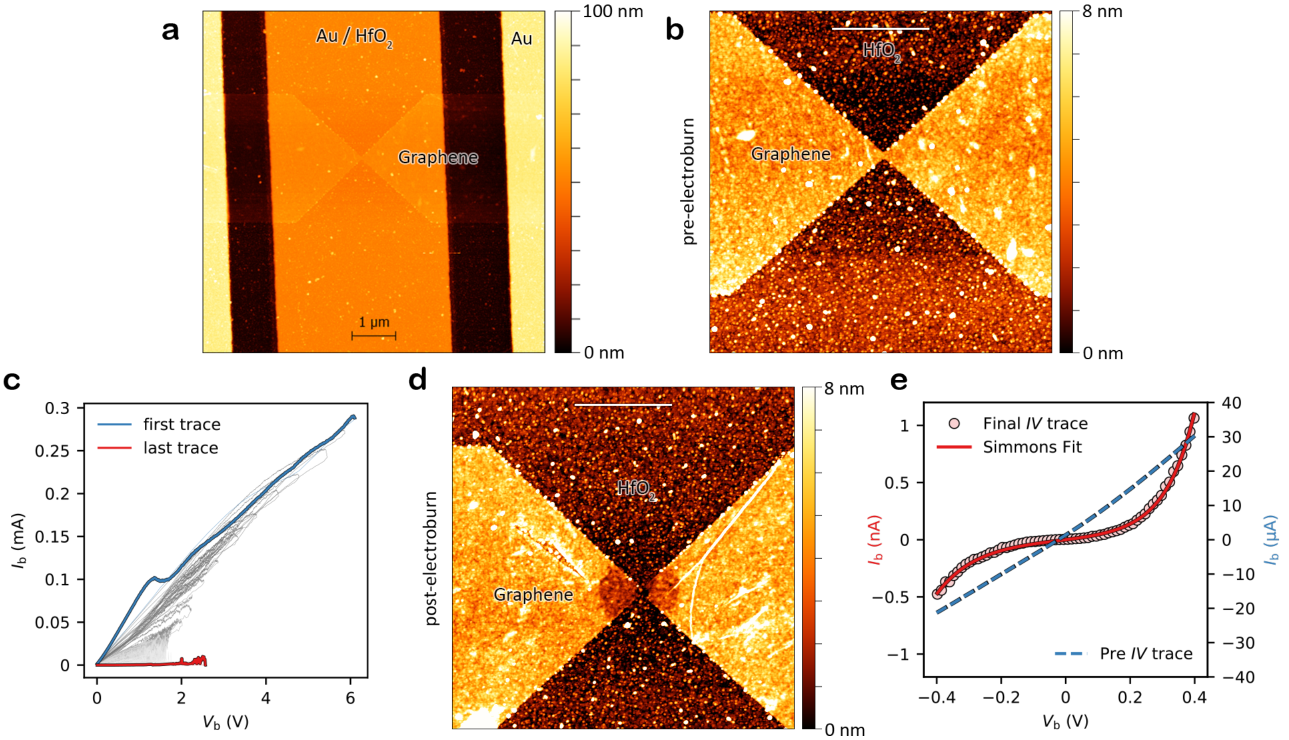

Supplementary Figure 1. Device fabrication. **a** AFM height image of a device pre-electroburn showing metal contacts for source, drain and gate. **b** A zoom of the graphene constriction in **a**. RMS-roughness: 1.75 nm (graphene), 1.95 nm (HfO_2). **c** Typical feedback-controlled electroburning traces of a graphene nanoribbon. **d** AFM image of an electroburned constriction. A cleaning circle is observed due to Joule heating during the process: high temperature occurring in the central region during the electroburning procedure effectively removes the contamination. RMS-roughness: 1.52 nm (graphene), 1.89 nm (HfO_2), 0.82 nm (Joule-heated part). **e** *IV* traces before and after electroburning, and the fit to the Simmons model.[1–3] All scale bars are 1 μm.

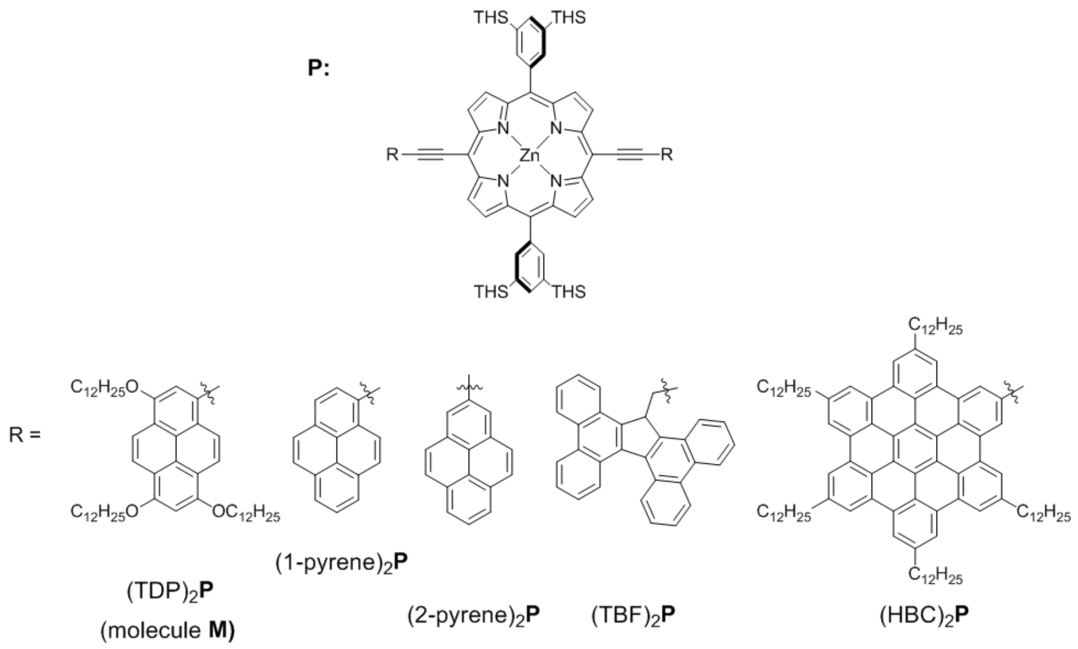

Supplementary Figure 2. Chemical structures of molecules deposited on graphene nanogaps. THS: trihexylsilyl.

Supplementary Note 1 Chemical structures

The chemical structures of the five molecules deposited from solution to form single-molecule transistors are given in Supplementary Figure 2. The porphyrin central unit (**P**) is functionalised with different groups designed to act as π-anchors, interfacing the molecule with the graphene electrodes. The synthesis of all five compounds has been reported previously.[4] $(TDP)_2$**P** is molecule **M**, and is the molecule in devices **A**-**D** in the main text, and device **E** in Section Supplementary Note 6. Devices **F**-**N** are displayed in Section Supplementary Note 7 and contain either one of $(1\text{-pyrene})_2$**P**, $(2\text{-pyrene})_2$**P**, $(TBF)_2$**P** or $(HBC)_2$**P**.

As stated in the main body of this work, during the oxidation of these compounds the charge density is predominantly withdrawn from both the anchor groups and the porphyrin ring, as opposed to the aryl side groups. This is shown in Supplementary Figure 3 which shows the single-particle molecular orbitals in various charge states most likely involved in the investigated resonant charge transport (where N corresponds to the neutral molecular species).

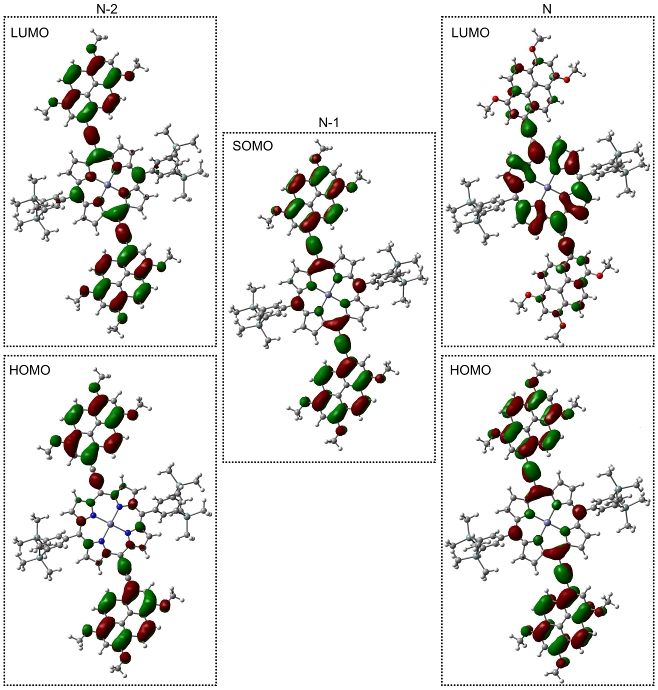

Supplementary Figure 3. Schematic plots of the frontier molecular orbitals calculated in Gaussian09[5] using B3LYP functional and 6-31G(d) basis set.

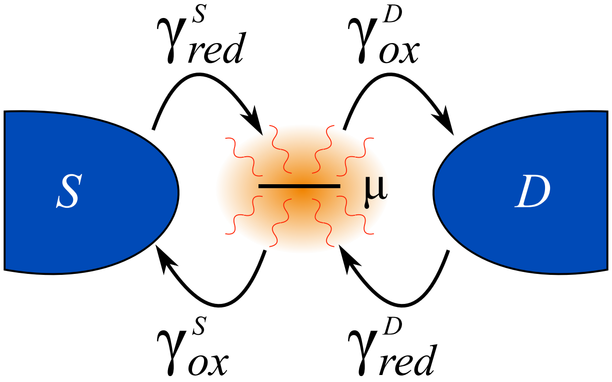

Supplementary Figure 4. Schematic of the theoretical model used here. The arrows illustrate the direction of various electron hopping processes.

Supplementary Note 2 Theory

In this section we outline the theoretical approach used to describe the transport properties of the molecular junctions. We describe the molecular structure within the junction as a single 'site' that is coupled to two (source S, and drain D) electrodes and is additionally interacting with a collection of (molecular and broader environmental) vibrational modes, as schematically pictured in Supplementary Figure 4. The molecular system is modelled as a single energy level with the energy μ (which depends on the gate voltage V_g). As described in the main body of this work, we assume that the vibrational modes interacting with the molecular energy level can be found in their thermal equilibrium state at all times.

Reduction and oxidation rates

As discussed in the main body of this work, we model the overall charge transport as a sequence of electron hoppings (oxidations and reductions) at the source and drain interfaces. We denote the rates of oxidation and reduction processes as γ^l_{ox} and γ^l_{red}, respectively, the molecular electrode couplings are given by Γ_l, where $l = S,D$. The reduction and oxidation rates at the source and drain interfaces are given by:

$$\gamma^l_{red} = (2-\Omega)\frac{\Gamma_l}{\hbar}\int_{-\infty}^{\infty} \mathrm{d}\varepsilon f_l(\varepsilon)\, k_{red}(\varepsilon)\ ; \qquad \text{(Supplementary Equation 1)}$$

$$\gamma^l_{ox} = (1+\Omega)\frac{\Gamma_l}{\hbar}\int_{-\infty}^{\infty} \mathrm{d}\varepsilon [1-f_l(\varepsilon)]\, k_{ox}(\varepsilon)\ , \qquad \text{(Supplementary Equation 2)}$$

respectively, as given in the main body of this work. In the above, $f_l(\varepsilon)$ denotes the Fermi-Dirac distribution: $f_l(\varepsilon) = (\exp[(\varepsilon-\mu_l)/k_B T]+1)^{-1}$ where μ_l is the chemical potential of the lead l. The parameter Ω depends on the degeneracy of the molecular electronic level and takes the value of 0 in the case of even N and $\Omega = 1$ otherwise. To understand the physical meaning of the $(2-\Omega)$ and $(1+\Omega)$ factors let us consider a system which (for a given V_g) is found in an even-N charge state at zero bias. As V_b is increased, the resonant transport (charging and de-charging of the molecule) becomes possible. Since the $N+1$ charge state is doubly degenerate (it possesses an odd number of electrons), the reduction rate is twice what would have been in the absence of this degeneracy – both spin-up and spin-down electrons can charge the molecule. The efficiency of an electron hopping off the molecule, on the other hand, is not affected since the N charge state is singly-degenerate. The opposite is true for an odd N.

The physical meaning of Supplementary Equation 1 and Supplementary Equation 2 is as follows. They describe the effective hopping rate between a donor and an acceptor system, one of which is represented by a single (molecular) energy level with the other one comprising a continuum of energy levels. To obtain an effective hopping rate, one must therefore integrate over this continuum of energy levels (each of which can act as a donor/acceptor) weighted by the Fermi distribution [or $1-f_l(\varepsilon)$] which describes the population of the above donor [acceptor] levels.

In Supplementary Equation 1, Supplementary Equation 2, $k_{red}(\varepsilon)$ and $k_{ox}(\varepsilon)$ are the molecular density of states (DOS), and are given by:[6]

$$k_{red}(\varepsilon) = \frac{1}{\pi}\,\mathrm{Re}\left[\int_0^\infty \mathrm{d}t\; e^{+\mathrm{i}(\varepsilon-\mu)t/\hbar} e^{-t/\tau} B(t)\right]\;; \qquad \text{(Supplementary Equation 3)}$$

$$k_{ox}(\varepsilon) = \frac{1}{\pi}\,\mathrm{Re}\left[\int_0^\infty \mathrm{d}t\; e^{-\mathrm{i}(\varepsilon-\mu)t/\hbar} e^{-t/\tau} B(t)\right]\;. \qquad \text{(Supplementary Equation 4)}$$

The above rates were derived using a second-order quantum master equation in the polaron-transformed frame. In order to go beyond the conventional Born approximation (which does not capture the lifetime broadening) the free system evolution was replaced with an effective one obtained through the equations of motion, see Ref.[6] for details. Therein, $\Gamma_l = 2\pi|V_l|^2\rho_l$ where V_l is the molecule-lead coupling strength (with the electrode l), ρ_l is the constant density of states in the lead l (wide-band approximation), and $\tau^{-1} = (\Gamma_S + \Gamma_D)/2\hbar$ is the lifetime of the molecular energy level. Overall, the damping term $e^{-t/\tau}$ induces lifetime broadening in our description (the origins of which can be traced back to the uncertainty principle which relates the uncertainty in energy of a quantum state to its lifetime). Finally, $B(t)$ is the phononic correlation function:

$$B(t) = \exp\left[\int_0^\infty \mathrm{d}\omega \frac{\mathcal{J}(\omega)}{\omega^2}\left(\coth\left(\frac{\beta\omega}{2}\right)\times\left(\cos\omega t - 1\right) - \mathrm{i}\sin\omega t\right)\right]\;, \qquad \text{(Supplementary Equation 5)}$$

where $\mathcal{J}(\omega)$ is the phononic spectral density which describes the distribution of the vibrational modes weighted by the strength of the electron-vibrational coupling. It is formally defined as:

$$\mathcal{J}(\omega) = \sum_q |g_q|^2 \delta(\omega - \omega_q)\;, \qquad \text{(Supplementary Equation 6)}$$

where g_q is the strength of the coupling between the molecular electronic energy level and the mode with frequency ω_q. The phononic correlation function $B(t)$ describes the nuclear (vibrational) dynamics accompanying the non-adiabatic electron transfer. Semi-classically, it can be understood as a time-dependent Franck-Condon factor, see Ref.[7] for a detailed discussion.

Rate Equation model

In the present case, the overall quantum master equation describing the dynamics of the system pictured in Supplementary Figure 4 can be reduced to a simpler rate equation model for the population of the empty P_N and charged molecular state P_{N+1}:[6]

$$\frac{\mathrm{d}P_N}{\mathrm{d}t} = -\left(\gamma^S_{red} + \gamma^D_{red}\right)P_N + \left(\gamma^S_{ox} + \gamma^D_{ox}\right)P_{N+1}\;; \qquad \text{(Supplementary Equation 7)}$$

$$\frac{\mathrm{d}P_{N+1}}{\mathrm{d}t} = -\left(\gamma^S_{ox} + \gamma^D_{ox}\right)P_{N+1} + \left(\gamma^S_{red} + \gamma^D_{red}\right)P_N\;. \qquad \text{(Supplementary Equation 8)}$$

We solve the above in the steady-state limit: $\frac{\mathrm{d}P_N}{\mathrm{d}t} = \frac{\mathrm{d}P_{N+1}}{\mathrm{d}t} = 0$. This yields:

$$P_N = \frac{\gamma^S_{ox} + \gamma^D_{ox}}{\gamma^S_{red} + \gamma^D_{red} + \gamma^S_{ox} + \gamma^D_{ox}}\;; \qquad \text{(Supplementary Equation 9)}$$

$$P_{N+1} = \frac{\gamma^S_{red} + \gamma^D_{red}}{\gamma^S_{red} + \gamma^D_{red} + \gamma^S_{ox} + \gamma^D_{ox}}\;. \qquad \text{(Supplementary Equation 10)}$$

The overall current is given by:

$$I = |e|\left(\gamma^D_{red}P_N - \gamma^D_{ox}P_{N+1}\right)\;, \qquad \text{(Supplementary Equation 11)}$$

which gives the expression given in the main body of this work:

$$I = |e| \, \frac{\gamma_{red}^{D}\gamma_{ox}^{S} - \gamma_{red}^{S}\gamma_{ox}^{D}}{\gamma_{red}^{S} + \gamma_{red}^{D} + \gamma_{ox}^{S} + \gamma_{ox}^{D}} \; . \qquad \text{(Supplementary Equation 12)}$$

Origin of Ω factors

To additionally justify the presence of the Ω prefactors in Eqs. (2) and (3) in the main body of this work let us consider the $N/N+1$ transition in detail (where N corresponds to a non-degenerate neutral charge state with an overall spin of zero). The set of rate equations for this scenario should account for the populations of three possible states: the non-degenerate N charge state (for simplicity referred to as P_0), and two degenerate $N+1$ charge state with an overall spin of $(+1/2)$ and $(-1/2)$, denoted by $P_\uparrow$ and $P_\downarrow$, respectively. Then, this set of rate equations can be compactly written as:

$$\frac{\mathrm{d}}{\mathrm{d}t}\begin{pmatrix} P_0 \\ P_\uparrow \\ P_\downarrow \end{pmatrix} = \begin{pmatrix} -2\gamma & \bar{\gamma} & \bar{\gamma} \\ \gamma & -\bar{\gamma} & 0 \\ \gamma & 0 & -\bar{\gamma} \end{pmatrix} \begin{pmatrix} P_0 \\ P_\uparrow \\ P_\downarrow \end{pmatrix} \; , \qquad \text{(Supplementary Equation 13)}$$

where γ is the overall reduction rate (for the spin-up *or* spin-down electrons):

$$\gamma = \sum_{l=S,D} \gamma_l = \sum_{l=S,D} \frac{\Gamma_l}{\hbar} \int \mathrm{d}\varepsilon f_l(\varepsilon) k_{red}(\varepsilon) \; , \qquad \text{(Supplementary Equation 14)}$$

and $\bar{\gamma}$ is the overall oxidation rate (again for the spin-up *or* spin-down electrons):

$$\bar{\gamma} = \sum_{l=S,D} \bar{\gamma}_l = \sum_{l=S,D} \frac{\Gamma_l}{\hbar} \int \mathrm{d}\varepsilon [1 - f_l(\varepsilon)] k_{ox}(\varepsilon) \; . \qquad \text{(Supplementary Equation 15)}$$

We note that $P_0 + P_\uparrow + P_\downarrow = 1$ and that in the absence of magnetic field, $P_\uparrow = P_\downarrow$. The steady-state populations can now be easily found:

$$P_0 = \frac{\bar{\gamma}}{\bar{\gamma} + 2\gamma} \; ; \qquad \text{(Supplementary Equation 16)}$$

$$P_\uparrow = P_\downarrow = \frac{\gamma}{\bar{\gamma} + 2\gamma} \; . \qquad \text{(Supplementary Equation 17)}$$

The electric current can be found at either molecule-lead interface. Considering, for instance, the source interface, the electric current is given by:

$$I = e[2\gamma_S P_0 - \bar{\gamma}_S P_\uparrow - \bar{\gamma}_S P_\downarrow] \; , \qquad \text{(Supplementary Equation 18)}$$

which trivially leads to an expression equivalent to the one in the main body of this work (for $\Omega = 0$).

High-temperature limit: Marcus theory

A number of simplifications to the above expressions can be made in the high-temperature limit.[6] Firstly, we note that the damping term $e^{-t/\tau}$ in Supplementary Equation 3, Supplementary Equation 4 induces only broadening of the *IV* characteristics. The broadening of the Fermi distributions in the leads will have the same effect on the transport properties of the junction, and we can therefore ignore this damping factor for $\tau^{-1} \ll k_\mathrm{B}T$. Secondly, we can take the high-temperature limits in the phononic correlation function in (Supplementary Equation 5): $\coth(\omega/(2k_\mathrm{B}T)) \approx 2k_\mathrm{B}T/\omega$; $\sin(\omega t) \approx \omega t$, and $\cos(\omega t) - 1 \approx -\omega^2 t^2/2$. This yields

$$B(t) \approx \exp\left(-\lambda t^2/\beta - \mathrm{i}\lambda t\right) \; , \qquad \text{(Supplementary Equation 19)}$$

where

$$\lambda = \int_0^\infty \mathrm{d}\omega \; \mathscr{J}(\omega)/\omega \qquad \text{(Supplementary Equation 20)}$$

is the reorganisation energy. The above assumptions give the well-known Marcus expressions[8–10] for the energy-dependent hopping rates:

$$k^{l}_{red/ox}(\varepsilon) = \sqrt{\frac{1}{4\pi\lambda k_\mathrm{B}T}}\exp\left(-\frac{(\lambda \pm (\varepsilon - \mu))^2}{4\lambda k_\mathrm{B}T}\right) \; , \qquad \text{(Supplementary Equation 21)}$$

as discussed in the main body of this work, see Ref.[6] for details.

Supplementary Note 3 DFT calculations

Gaussian 09[5] was used to carry out geometry optimisations and frequency calculations for different charge states of the molecules studied; B3LYP/6-31G(d) functional/basis set combination was used. From the results it is possible to calculate *ab initio* values for electron-vibration couplings and generate a spectral density that accounts for the inner sphere reorganisation of the molecules we have studied in nanogaps. For each vibrational mode an electron-vibration coupling constant, Λ_q, can be obtained from

$$\Lambda_q = K\sqrt{\frac{\omega_q}{2\hbar}} , \qquad \text{(Supplementary Equation 22)}$$

where ω_q is the vibrational frequency of mode q, and K is the Duschinsky shift vector.[11] The shift vector is obtained from (Supplementary Equation 23)

$$K = (mL'V^{-1/2})^{\mathrm{T}}\,(B^{-1}x_0 - x_0') , \qquad \text{(Supplementary Equation 23)}$$

where x_0 and x_0' are optimised coordinates in final and initial states, respectively. L' is the $(3N \times 3N-6)$ normal mode matrix of the initial state outputted by Gaussian09, m is a $(3N \times 3N)$ matrix that contains the masses of the atoms along the diagonal three times, and V is a $(3N-6 \times 3N-6)$ matrix that contains the reduced masses of each vibration along the diagonal. The purpose of m and V is to mass-weight the normal mode matrix; B is the axis-switching matrix.[12]

The total inner sphere reorganisation energy, λ_i, obtained by this method is given by the sum over the reorganisation energy for each mode:

$$\lambda_i = \hbar\sum_q \omega_q S_q , \qquad \text{(Supplementary Equation 24)}$$

where $S_q = \Lambda_q^2$ is the Huang-Rhys parameter.

Molecular structures

For the DFT work presented here, the molecular structures have been simplified in two ways. Firstly, the 3,5-bis(trihexylsilyl)phenyl groups (used to enhance the solubility of our porphyrins) have been replaced by hydrogens. Secondly, the long alkoxy chains on the anchors of $(TDP)_2P$ and $(HBC)_2P$ have also been replaced by -OH groups. The 3,5-bis(trihexylsilyl)phenyl groups are twisted to an angle of 70° with respect to the porphyrin ring, and therefore only weak conjugation will be present between the π-systems of these groups. The electrons of the alkyl chains are localised in σ-bonds. Therefore the substitutions will have a minimal influence on the electronic properties of the porphyrins, whilst greatly reducing the computational cost of the *ab initio* calculations presented in the following sections. One additional step was added to the analysis – it was assumed that the anchor groups of the molecule are bound to graphene and this 'clamping' suppresses vibrations that exhibit large out-of-plane motions of the anchor groups. Therefore the vibrations that contain large out-of-plane motions of the anchor atoms ($z > 0.02$ Å amu$^{1/2}$) are filtered from the vibrational analysis.[13] The sliding energy (the change in energy when moving the molecule parallel to the substrate) is significantly lower than the change in energy associated with moving the molecule in the perpendicular direction (DFT calculations suggest the sliding energy is around 1% of the binding energy in the perpendicular direction) and therefore vibrations in which there are in-plane motions (e.g. x & y) are retained.[13,14] This filtering did not make a difference to the (pyrene-2)$_2$**P**, (HBC)$_2$**P**, (TBF)$_2$**P** coupling constants, however filters some vibrations from the analysis of (pyrene-1)$_2$**P** and (TDP)$_2$**P** as there are changes in the angle between the π-anchors and central porphyrin unit depending upon the charge state of the molecule.

Ab initio electron-vibration coupling constants

In Supplementary Figures 5 to 11 we plot the electron-vibration coupling constants of the above DFT calculations for the molecular compounds pictured in Supplementary Figure 2. Supplementary Table 1 shows the resulting (inner-sphere) reorganisation energies.

Anchor	λ_i $(N-1 \to N)$ (meV)	λ_i $(N \to N+1)$ (meV)
TDP	67	55
Pyrene-1	88	124
Pyrene-2	72	56
TBF	27	50
HBC	132	61

Supplementary Table 1. DFT-calculated reorganisation energies for various charge transitions of the zinc-porphyrins coupled with anchors used in the experiments. N is the number of electrons on the molecule in the neutral state.

Molecule $\mathbf{M}-(\mathrm{TDP})_2\mathbf{P}$

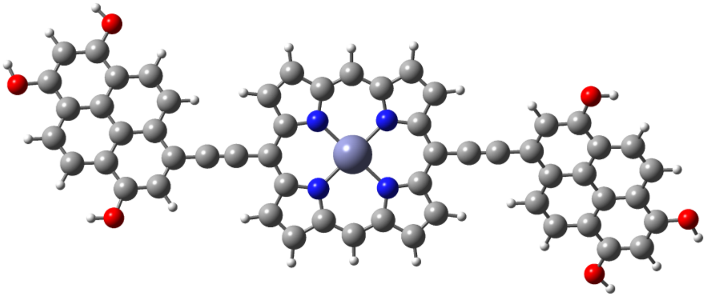

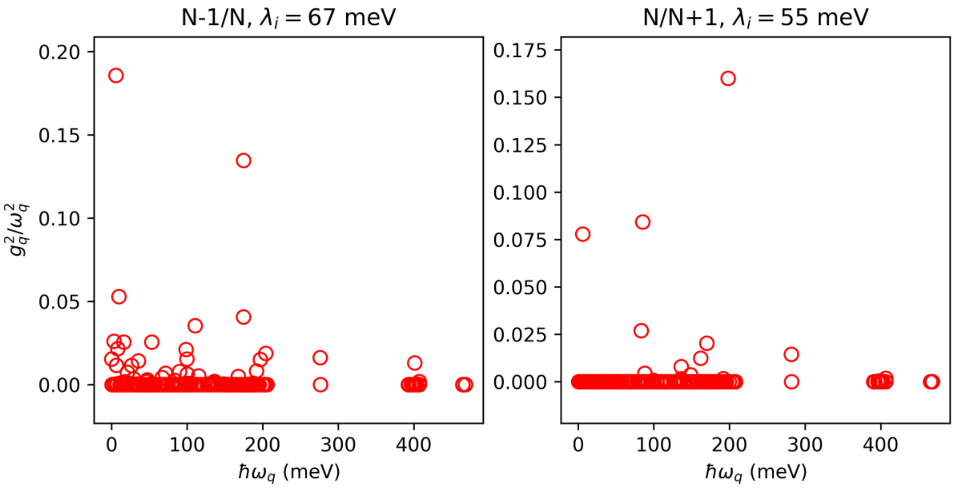

Supplementary Figure 5. $(\mathrm{TDP})_2\mathbf{P}$ electron-phonon coupling constants. Top: optimised neutral geometry of molecule $\mathbf{M}-(\mathrm{TDP})_2\mathbf{P}$. Bottom: electron-phonon coupling constants, and inner reorganisation energies for the $N-1/N$ and $N/N+1$ transitions.

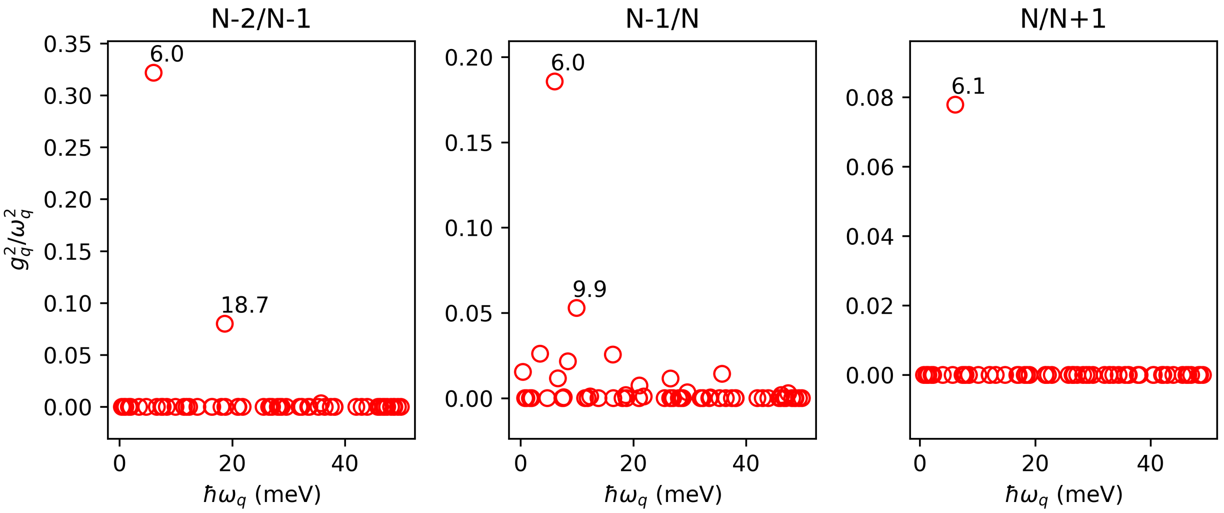

Supplementary Figure 6. Electron-phonon couplings constants below 50 meV, showing the presence of a single mode around 6 meV that is strongly coupled to the $N-2/N-1$, $N-1/N$, and $N/N+1$ transitions.

(1-pyrene)$_2$**P**

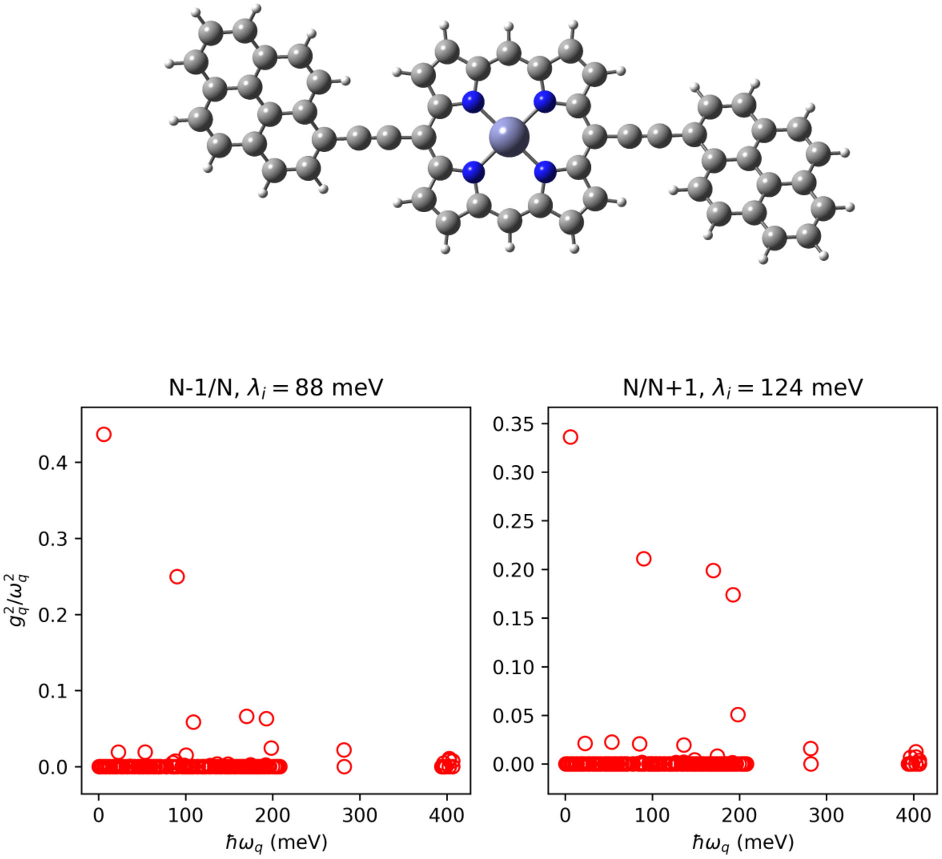

Supplementary Figure 7. (1-pyrene)$_2$**P** electron-phonon coupling constants. Top: optimised neutral geometry of (1-pyrene)$_2$**P**. Bottom: electron-phonon coupling constants, and inner reorganisation energies for the $N-1/N$ and $N/N+1$ transitions.

(2-pyrene)$_2$**P**

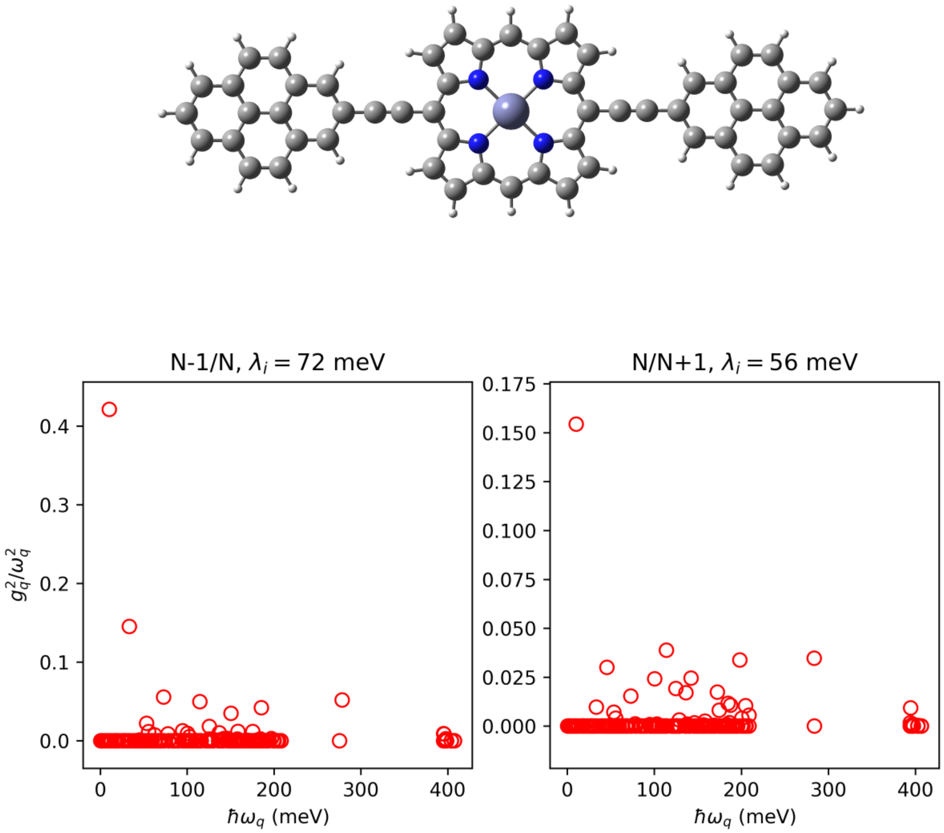

Supplementary Figure 8. (2-pyrene)$_2$**P** electron-phonon coupling constants. Top: optimised neutral geometry of (2-pyrene)$_2$**P**. Bottom: electron-phonon coupling constants, and inner reorganisation energies for the $N-1/N$ and $N/N+1$ transitions.

$(HBC)_2$**P**

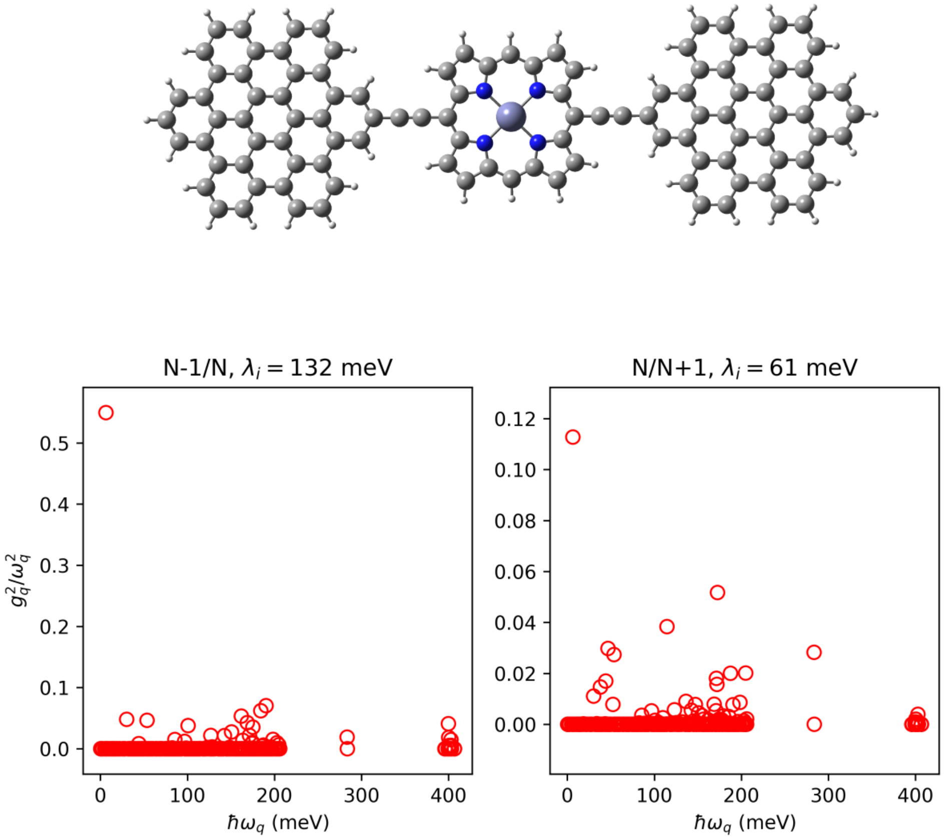

Supplementary Figure 9. $(HBC)_2$**P** electron-phonon coupling constants. Top: optimised neutral geometry of $(HBC)_2$**P**. Bottom: electron-phonon coupling constants, and inner reorganisation energies for the $N-1/N$ and $N/N+1$ transitions

(TBF)$_2$**P**

In the (TBF)$_2$**P** structure there is an sp^3-hybridised carbon between the acetylene and the π-anchor, and the anchor rotates with respect to the porphyrin upon geometry optimisations of different charge states. This leads to unphysically large values of Λ_q and λ_i. Supplementary Figure 10 shows the HOMO/LUMO are localised upon the porphyrin moiety, therefore we assume geometric changes will also be localised to this portion of the molecule, and to calculate the electron-phonon couplings of the charge transitions of (TBF)$_2$**P** we can replace the TBF anchors with hydrogen atoms.

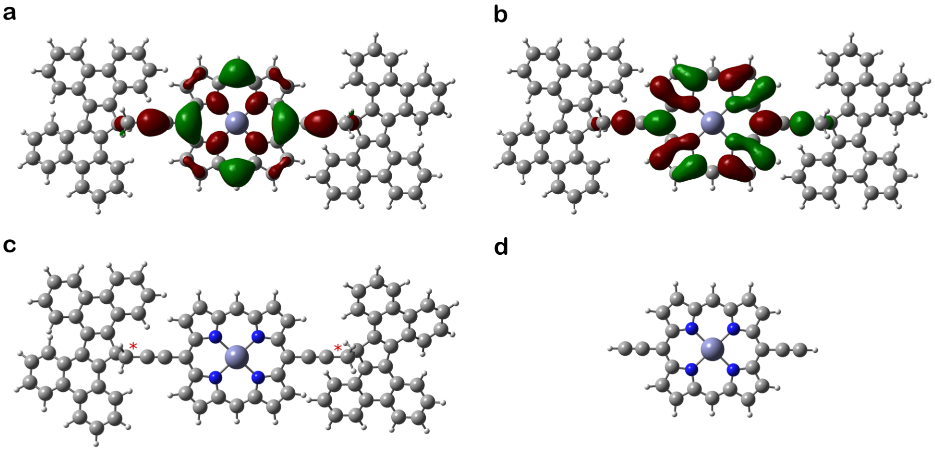

Supplementary Figure 10. Simplification of (TBF)$_2$**P**. **a** HOMO and **b** LUMO of neutral (TBF)$_2$**P**, **c** the sp^3-carbons are highlighted by the red asterisks on the optimised neutral geometry. **d** The optimised neutral geometry of truncated molecule used for calculating the electron-phonon couplings for (TBF)$_2$**P** transitions.

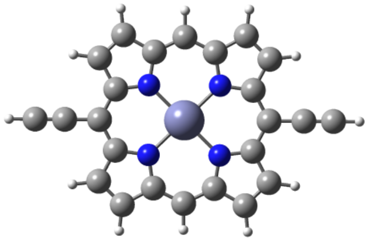

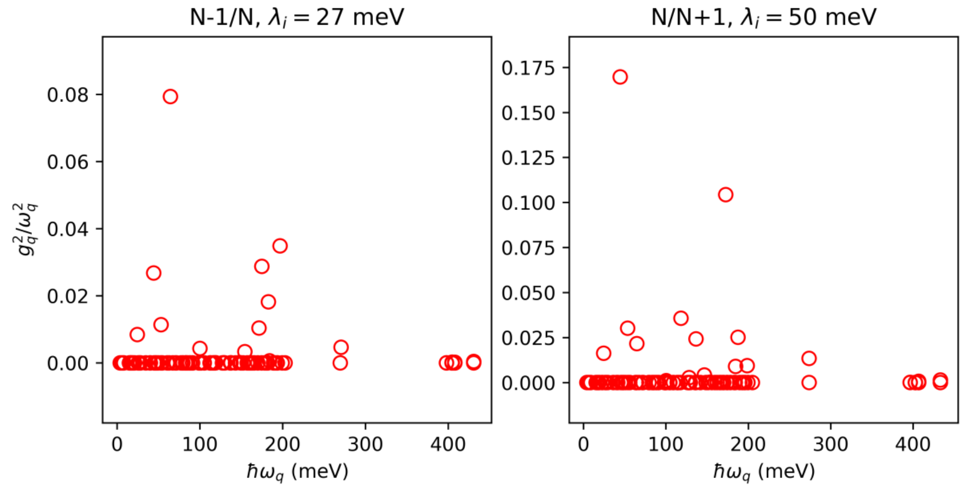

Supplementary Figure 11. $(TBF)_2$**P** electron-phonon coupling constants. Top: optimised neutral geometry of $(TBF)_2$**P**, electron-phonon coupling constants. Bottom: inner reorganisation energies for the $N-1/N$ and $N/N+1$ transitions.

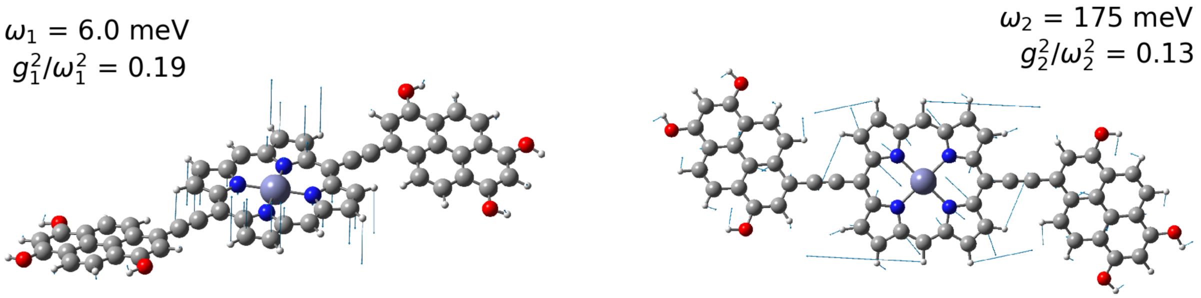

Supplementary Figure 12. Schematic representations of two vibrational modes (with frequencies of 6 and 175 meV) that are relatively strongly coupled to the electronic degrees of freedom in the (TDP)$_2$**P** structure.

Examples of vibrational modes

We finish this section by schematically plotting in Supplementary Figure 12 two relatively strongly coupled modes calculated for the $N-1/N$ transition of the molecule **M** [(TDP)$_2$**P**]. The low-frequency mode (at 6 meV) corresponds to a collective twist of the anchor groups with respect to the porphyrin core. The higher-frequency mode is a combination of various bond stretches, atom rockings and wagings. We note, however, that the exact nature of the considered vibrational modes is inconsequential to the conclusions of this work.

The effect of bulky side-groups

In the above, to make our calculations tractable, we have ignored the bulky side groups (shown in Section Supplementary Note 1). This can be justified by the fact that, as shown in Section Supplementary Note 1, the frontier orbitals have very little electron density on the aforementioned side groups. Consequently, the charging of the molecule is not expected to alter the equilibrium nuclear positions of atoms in these bulky side-groups.

Supplementary Note 4 Substrate reorganisation energy

As discussed in the main body of this work, besides the molecular vibrational modes, the molecular electronic energy level can be also coupled to the phononic modes of the substrate. Here, we limit ourselves to a phenomenological description of these interactions, and assume that they can be accounted for the following spectral density:

$$\mathscr{J}(\omega) = \frac{\lambda_o}{2}\left(\frac{\omega}{\omega_c}\right)^3 e^{-\omega/\omega_c}\ , \qquad \text{(Supplementary Equation 25)}$$

where λ_o is the reorganisation energy for this (outer-sphere) interaction, and ω_c is the cut-off frequency. The average frequency is given by $\langle\omega\rangle = 4\omega_c$.

One should anticipate that the cut-off frequency an intrinsic property of the substrate while the reorganisation energy (which quantifies the molecule-substrate vibrational coupling strength) depends on the orientation and position of the zinc-porphyrin on the substrate and should be expected to vary between different devices.

The aforementioned (surface-induced) reorganisation energy can be estimated (for a given molecule-surface distance d) as:[15,16]

$$\frac{\lambda_o}{2} \approx \left(\frac{\varepsilon_{r0}-1}{\varepsilon_{r0}+1} - \frac{\varepsilon_{r\infty}-1}{\varepsilon_{r\infty}+1}\right)\frac{\tilde{Q}_F^2}{16\pi\varepsilon_0 d} + \left(\frac{4\varepsilon_{r0}}{(\varepsilon_{r0}+1)^2} - \frac{4\varepsilon_{r\infty}}{(\varepsilon_{r\infty}+1)^2}\right)\frac{\tilde{Q}_M^2}{16\pi\varepsilon_0(d+a_{tot})} \qquad \text{(Supplementary Equation 26)}$$

In the above, ε_{r0} is the static dielectric constant of the insulator (SiO_2) film: $\varepsilon_{r0} = 3.9$; $\varepsilon_{r\infty}$ is the optical dielectric constant: $\varepsilon_{r\infty} = 2.3$. $\tilde{Q}_{F,M}$ are the effective charges (fractions of the electric charge e) defined in (B8) of Ref.[16] which depend on the porphyrin-substrate separation, d. We approximate the porphyrin as a 7×7 Å square with a uniformly distributed charge (assuming the additional charge density is not localised on the anchor groups). Furthermore, a_{tot} is the total width of the insulator film, $a_{tot} = 300$ nm. Consequently, the second term on the right-hand side of (Supplementary Equation 26) can be largely ignored.

We estimate that the porphyrin-substrate distance is roughly $d \sim 0.6$ nm (as inferred from the AFM measurements of the porphyrin compounds deposited on highly oriented pyrolytic graphite, see SI of Ref.[4]). Using formula (Supplementary Equation 26) we therefore estimate the outer-sphere reorganisation energy as $\lambda_o \sim 200$ meV.

When modelling the 77 K data, we set ω_c to 25 meV which results in an excellent match of the model used here to the experimental data for all considered devices (*vide infra*), as well as yielding λ_o values which correspond to porphyrin-substrate distances of between roughly 0.5 and 1 nm (in agreement with the AFM studies), see Section Supplementary Note 7.

We note however that the effectiveness of our model (quality of the fit of the *IV* characteristics) is largely unaffected by the exact choice of ω_c (within the range of roughly 10 to 50 meV). In Supplementary Figure 13 we compare the experimental and fitted *IV* characteristics for different values of ω_c. A reasonable qualitative agreement can be reached regardless of the exact value of ω_c. Quantitatively, the model used here performs best for $\omega_c \sim 25$ meV. This is further shown in Supplementary Figure 14 where we compare the experimental and calculated stability diagrams (for the molecule-lead couplings and λ_o extracted in Supplementary Figure 13) again for different values of ω_c.

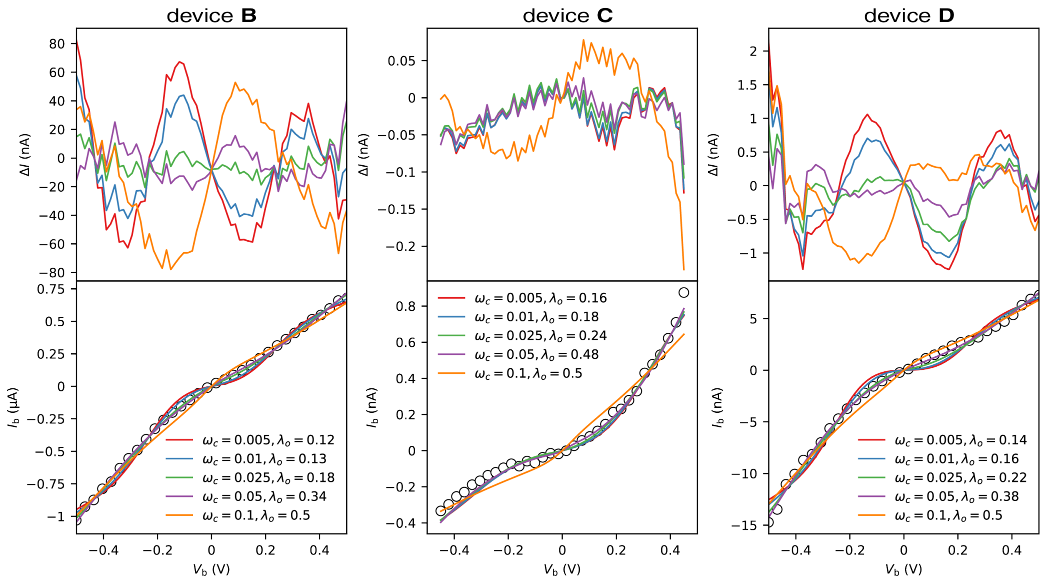

Supplementary Figure 13. Comparison of fits for different ω_c values. Bottom: Experimental and fitted *IV* characteristics for devices **B**–**D**. The latter were obtained with fixed values of ω_c (as specified). Top: differences between the experimental and fitted *IV* characteristics. All extracted values in units of eV.

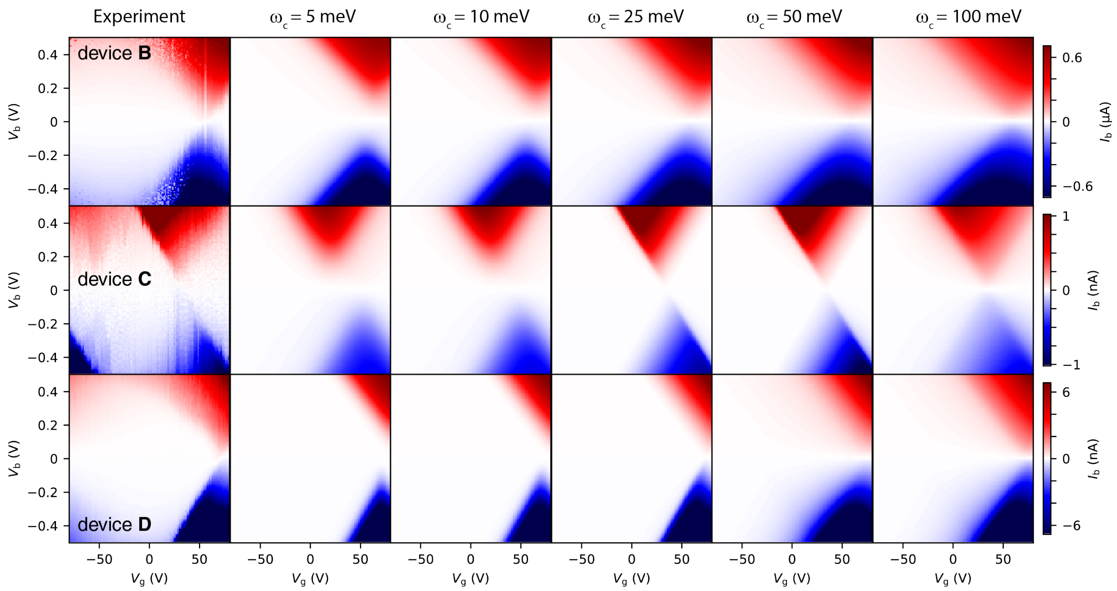

Supplementary Figure 14. Experimental and calculated stability diagrams for devices **B**–**D**. The latter were obtained through fitting of the *IV* characteristics with the values of ω_c between 5 and 100 meV, as specified.

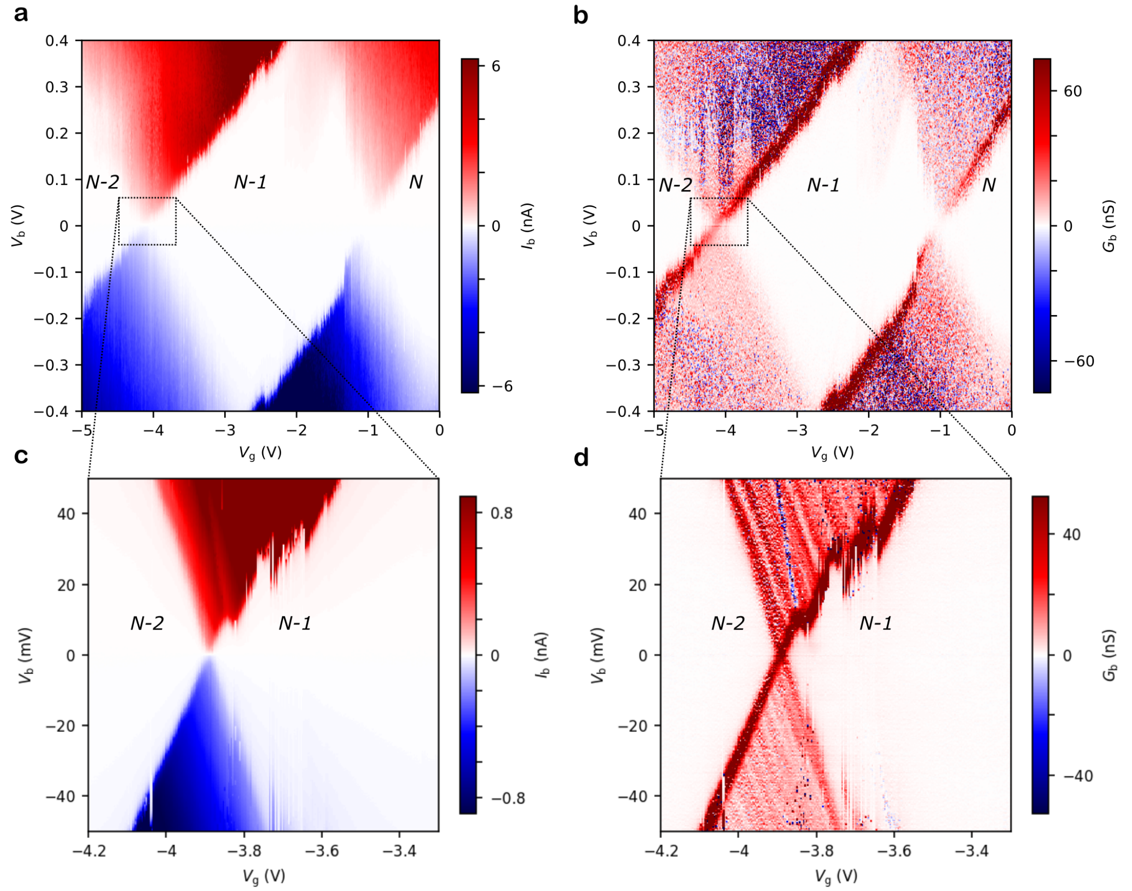

Supplementary Figure 15. Full stability diagram for device **A**. **a,c** Stability diagrams and **b,d** conductance maps for device **A** measured at $T = 7$ K. N denotes the neutral molecular structure.

Supplementary Note 5 Supporting data for device A

Supplementary Figure 15 shows the full stability diagram for device **A** measured within a larger bias and gate voltage range at $T = 7$ K. The charge states are assigned using the method outlined in Ref [17] The highest-current corners of the resonant transport regions are the bottom-left of $N-1/N$ transition and the top-right of the $N-2/N-1$ charge transition. Supplementary Figure 15(d) clearly shows that the lowest-energy excited state lines run parallel to the edges of the Coulomb diamond. They are therefore very likely to be a result of the electron-vibrational interactions.

Furthermore, Supplementary Figure 16 shows the experimental, as well as calculated, zero-bias conductance of device **A** at $T = 5$ K. The two theoretical results were obtained with and without lifetime broadening respectively,[6,18] and using the parameter values extracted in the main body of this work. As can be seen in Supplementary Figure 16, the zero-bias conductance is overestimated by $\sim 30\%$ in the absence of lifetime broadening.

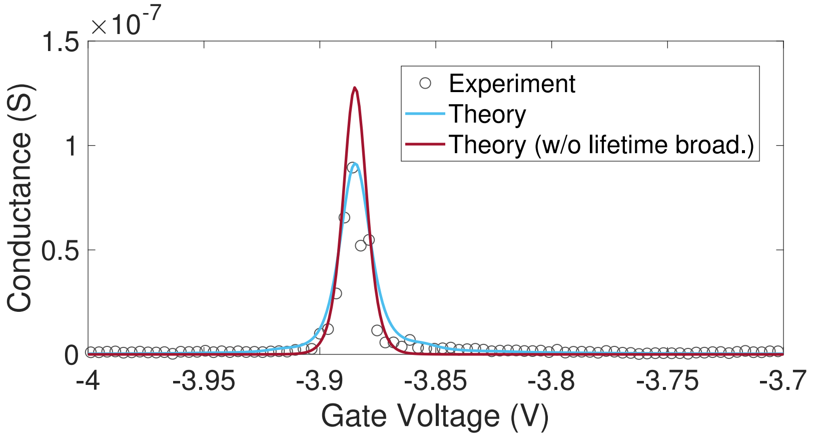

Supplementary Figure 16. Experimental and calculated zero-bias conductance of device **A** at $T = 5$ K.

Supplementary Note 6 Supporting 5 K data (device E)

Supplementary Figure 17 shows the conductance map (left) and the averaged conductance peaks (right) of device **E** which comprises the molecule **M** [$(TDP)_2$**P** structure]. The observed conductance peaks very well match those observed in device **A**, *c.f.* Figure 1(c) in the main body of this work, which confirms their molecular (vibrational) origin.

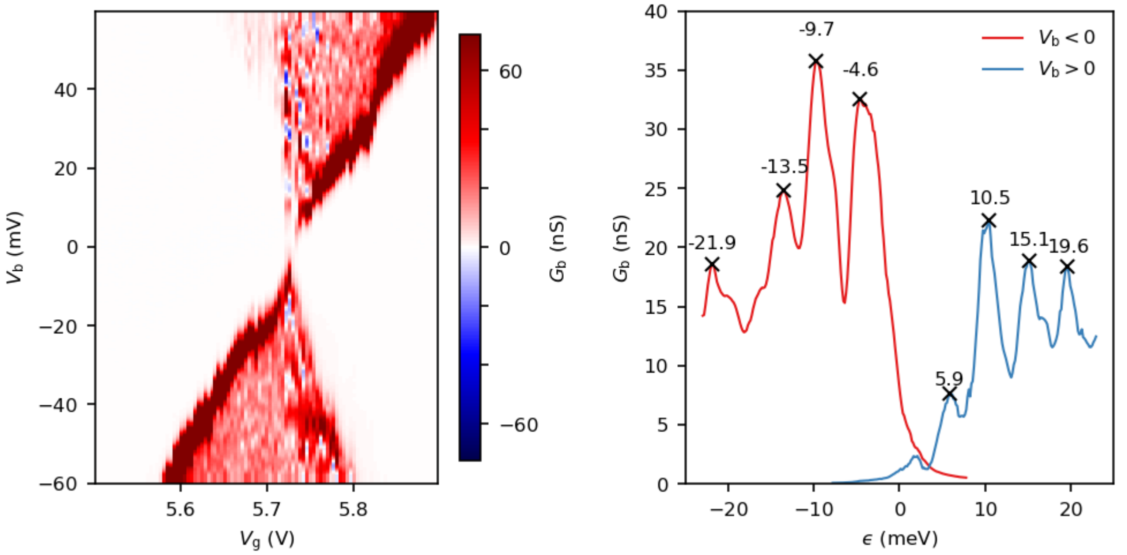

Supplementary Figure 17. Device **E**. Left: Conductance map of device **E** at 5 K, Right: the conductance of the resonant triangles as an average of the lines running parallel to the edge of the triangle. The modes correspond very well to those of device **A**.

Supplementary Note 7 Supporting 77 K data

In order to enable a more detailed comparison of the experimental and theoretically-predicted transport behaviour, in Supplementary Figures 18 to 20 we plot a number of experimental gate traces and compare them to those predicted by the quantum model and Marcus transport theories.

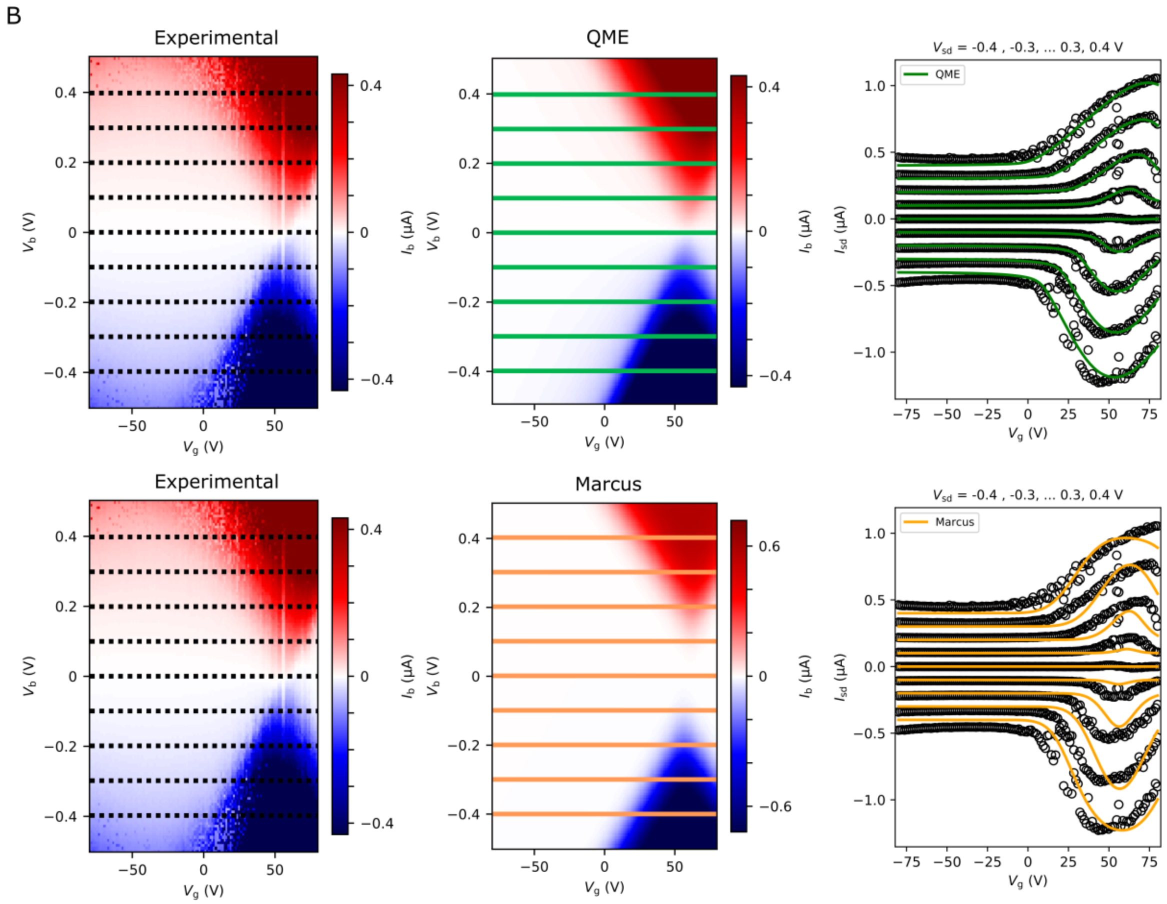

Supplementary Figure 18. Device **B**. Left: Experimental and theoretical stability diagrams at 77 K (from the main body of this work). Right: Comparison of experimental and theoretical gate traces.

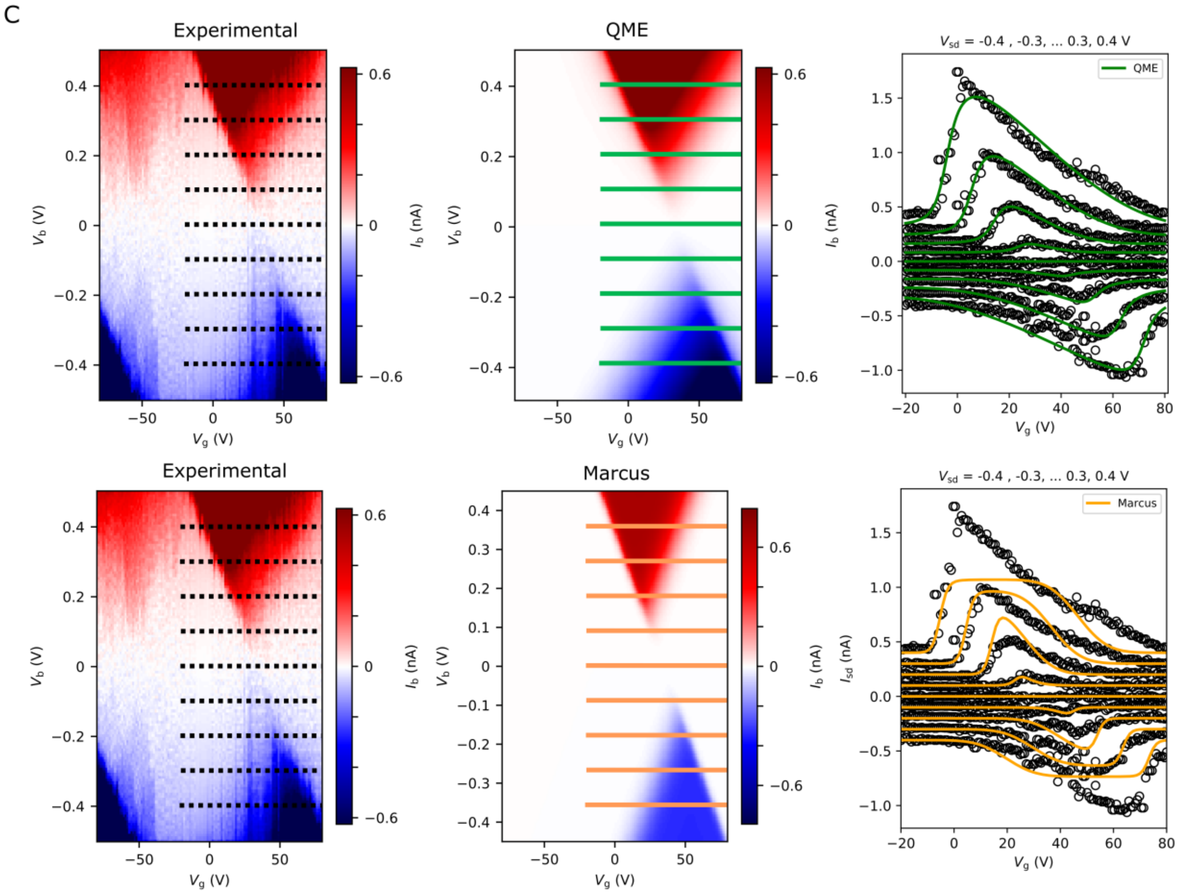

Supplementary Figure 19. Device **C**. Left: Experimental and theoretical stability diagrams at 77 K (from the main body of this work). Right: Comparison of experimental and theoretical gate traces.

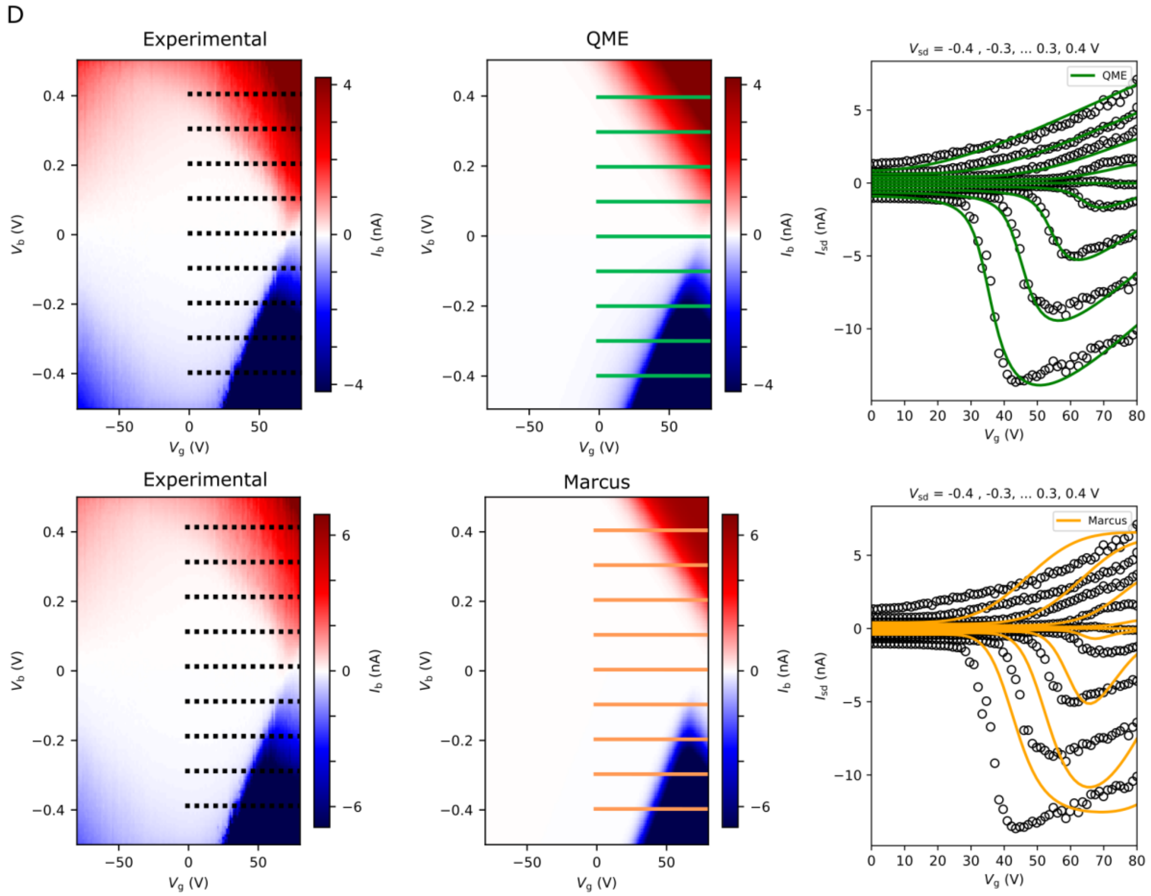

Supplementary Figure 20. Device **D**. Left: Experimental and theoretical stability diagrams at 77 K (from the main body of this work). Right: Comparison of experimental and theoretical gate traces.

Devices F-N

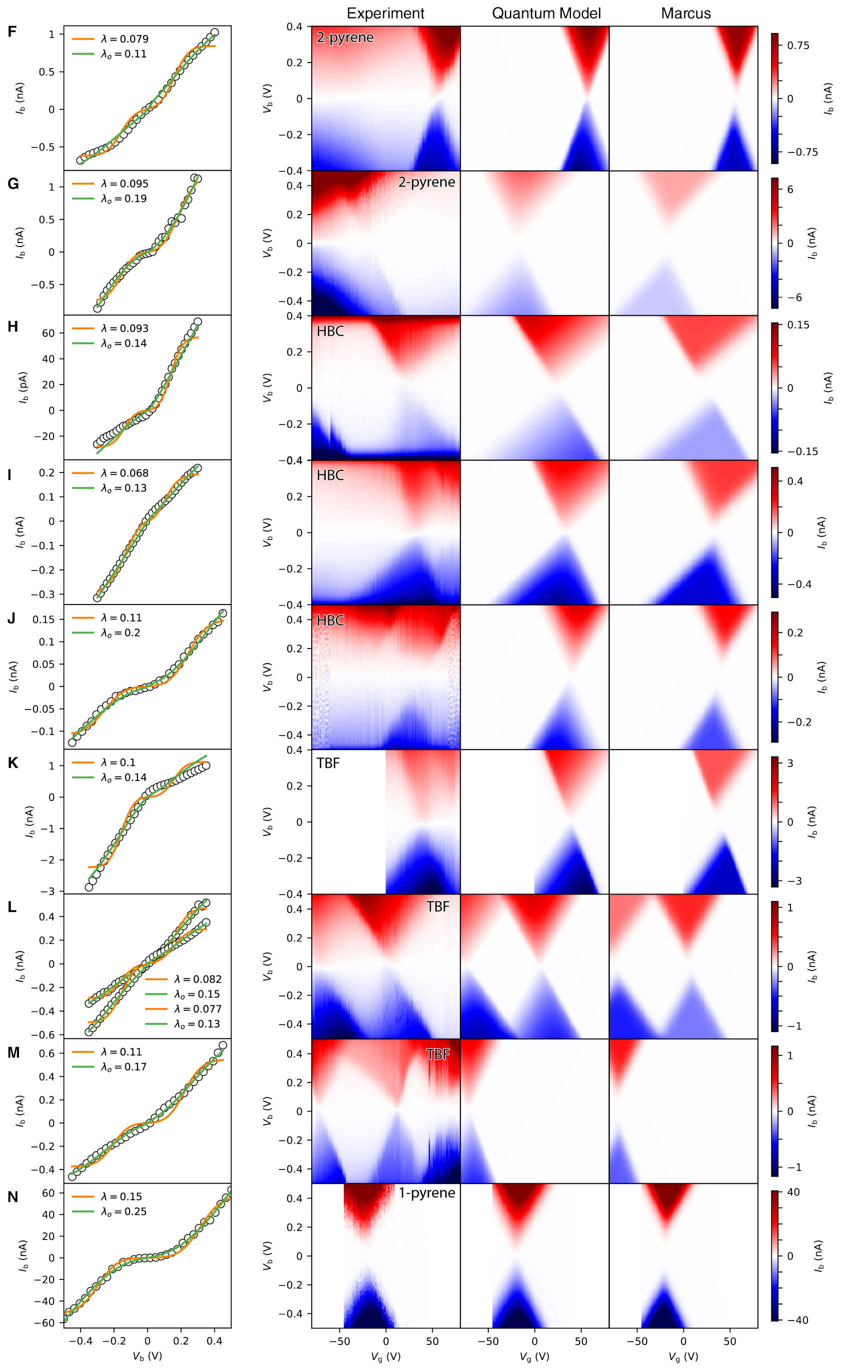

Supplementary Figure 21. Charge transport data and comparison of fits for devices **F–N**. Left: *IV* traces at resonance of the devices at 77 K (data from Ref. 1) and the fit to the Marcus model (orange) and the quantum model (green). Right: charge stability diagrams of the devices at 77 K, and the reconstructed stability diagrams from the fits on the left according the quantum model, or the Marcus model.

**device**	Molecule	Γ_S (eV)	Γ_D (eV)	λ_o (eV)	distance (Å)
**B**	(TDP)$_2$**P**	2.19×10^{-2}	5.65×10^{-3}	0.18	7.1
**C**	(TDP)$_2$**P**	4.33×10^{-6}	2.51×10^{-3}	0.24	5.1
**D**	(TDP)$_2$**P**	1.61×10^{-3}	5.28×10^{-5}	0.22	5.8
**F**	(2-pyrene)$_2$**P**	1.41×10^{-5}	5.43×10^{-6}	0.11	13
**G**	(2-pyrene)$_2$**P**	1.06×10^{-5}	7.26×10^{-5}	0.19	6.7
**H**	(HBC)$_2$**P**	3.32×10^{-7}	5.55×10^{-4}	0.14	9.7
**I**	(HBC)$_2$**P**	1.76×10^{-6}	1.06×10^{-5}	0.13	10.4
**J**	(HBC)$_2$**P**	3.44×10^{-6}	1.51×10^{-6}	0.20	6.3
**K**	(TBF)$_2$**P**	7.60×10^{-6}	3.04×10^{-3}	0.14	9.9
**L**	(TBF)$_2$**P**	2.57×10^{-6}	1.61×10^{-5}	0.13	9.3
		2.74×10^{-6}	2.04×10^{-5}	0.13	10.5
**M**	(TBF)$_2$**P**	2.99×10^{-6}	1.23×10^{-5}	0.17	7.7
**N**	(1-pyrene)$_2$**P**	7.60×10^{-4}	1.41×10^{-3}	0.25	4.8

Supplementary Table 2. Fitting parameters for all the 77 K devices (with $\omega_c = 25$ meV):

In the above, the distance of the porphyrin from the surface (SiO_2) was approximated in accordance with Refs.[15,16] (see Section Supplementary Note 4), by modelling the porphyrin as a 7×7 Å square with a uniformly distributed charge and an infinitely thick dielectric. The distances obtained from the fitted values of λ_o are plotted in Supplementary Figure 22.

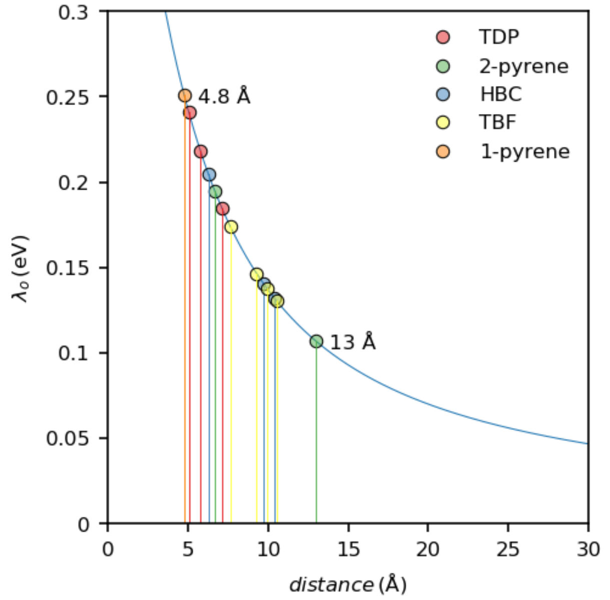

Supplementary Figure 22. Substrate reorganisation energy λ_o as a function of the porphyrin distance to the substrate. The porphyrin was modelled as a 7×7 Å square with a uniformly distributed charge. The extracted porphyrin-substrate distances are in agreement with the AFM studies of Limburg *et al.*[4]

Supplementary Note 8 Alternative intermediate-temperature models

As discussed in the main body of this work, Marcus theory (MT) fails to correctly describe the transport behaviour of considered molecular junctions at the liquid-nitrogen temperature, $T = 77$ K. Here, we attempt to account for the empirical data with two low-temperature extensions of the conventional Marcus theory.

Firstly, we consider the Low-Temperature Corrected MT (LTC-MT) derived in Ref.[6]. It is derived from the phononic correlation function in (Supplementary Equation 5) by retaining higher-order terms while taking the high-temperature limit. The molecular DOS are given by:[6]

$$k^{l}_{red/ox}(\varepsilon) = \frac{1}{\pi}\sqrt{\frac{\pi}{4\lambda k_{\mathrm{B}}T + \chi/3k_{\mathrm{B}}T}}\exp\left(-\frac{(\lambda \pm (\varepsilon-\mu))^2}{4\lambda k_{\mathrm{B}}T + \chi/3k_{\mathrm{B}}T}\right) , \qquad \text{(Supplementary Equation 27)}$$

where the low-temperature correction is introduced through the presence of additional parameter

$$\chi = \int_0^\infty \mathrm{d}\omega\ \omega \times \mathscr{J}(\omega) , \qquad \text{(Supplementary Equation 28)}$$

which accounts for the coupling to high-frequency vibrational modes for which the high-temperature assumption of the conventional Marcus theory is not justified.

Secondly, we shall make use of Marcus-Jortner theory.[19] It separates the low-frequency environmental coupling (which is treated as in the conventional Marcus theory with the reorganisation energy λ_o) and the high-frequency molecular modes. The latter are approximated by a single effective mode with frequency ω_0 which couples to the electronic degrees of freedom with the Huang-Rhys parameter S. The molecular DOS are then given by:[19]

$$k^{l}_{red/ox}(\varepsilon) = \frac{1}{\pi}\sqrt{\frac{\pi}{4\lambda k_{\mathrm{B}}T}}\sum_{m=0}^{\infty} e^{-S}\exp\frac{-\left(\lambda_o + m\omega_0 \pm (\varepsilon-\mu)\right)^2}{4\lambda_o k_{\mathrm{B}}T}\frac{S^m}{m!} \qquad \text{(Supplementary Equation 29)}$$

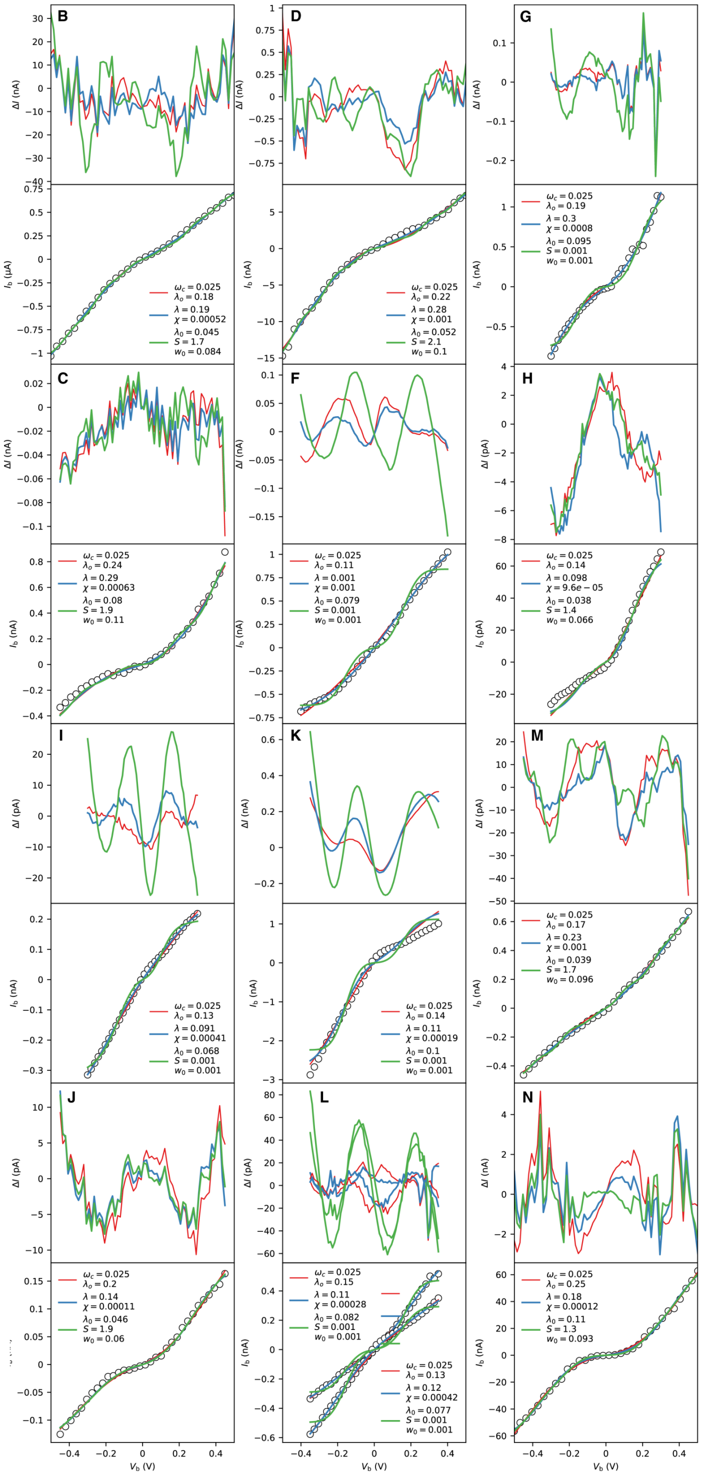

Supplementary Figure 23. Charge transport data and comparison of fits to intermediate-temperature models for devices **F**–**N**. Bottom: *IV* traces at resonance of devices at 77 K and the fit to the quantum model from the main body of this work (red) and the LTC-MT (blue), and Marcus-Jortner theory (green). Top: Differences between the fitted and experimental *IV* characteristics.

Supplementary Figure 23 shows the experimental IV traces on resonance as well as the theoretical fits to the quantum approach (from the main body of this work), LTC-MT and Marcus-Jortner theory. LTC-MT yields comparable fits to the (more sophisticated) quantum approach. On the other hand, the Marcus-Jortner approach tends to, in general, perform worse than the above methods as it gives rise to artefacts akin to those of the usual Marcus treatment. The artefacts are visible most clearly in 5 out of 12 devices - D, F, G, I, and L. Despite this, it still performs better than the conventional Marcus approach although at the cost of two additional fitting parameters. We then use

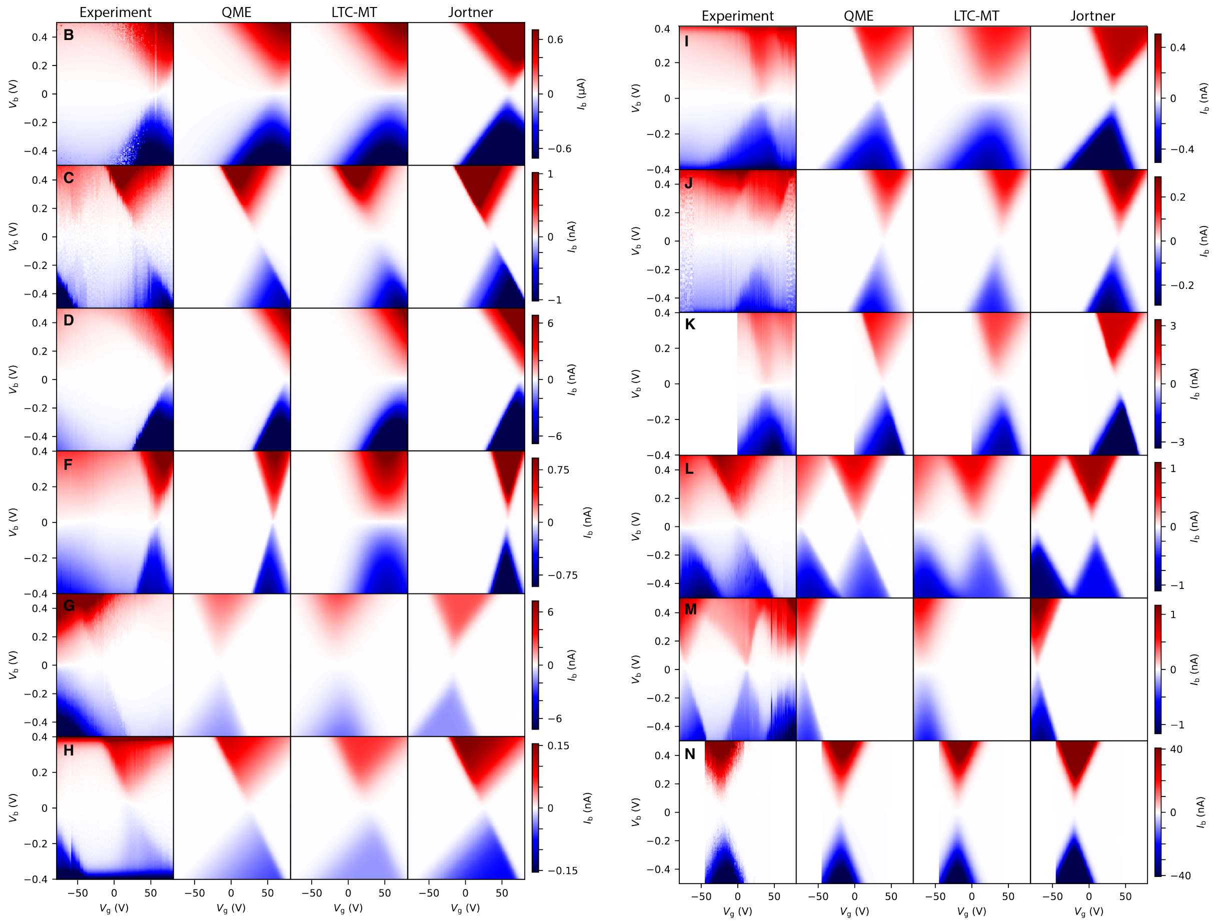

Supplementary Figure 24. Experimental and calculated stability diagrams for devices **B–D**, **F–M** at 77 K.

the parameters from these fits to reconstruct the full stability diagrams and compare these to experimental data, this is shown in Supplementary Figure 24. Despite reproducing the IV traces well, LTC-MT overestimates the degree of vibrationally-induced broadening of the IV characteristics off resonance, in agreement with earlier predictions.[6]. This can be seen most evidently in the stability diagrams of devices such as B, C, F, G, and H. The performance of Marcus-Jortner approach is comparable to that of the conventional Marcus theory as it again predicts early plateaus in the IV characteristics, *c.f.* Supplementary Figure 21. Overall, Marcus-Jortner and LTC-MT approaches both have drawbacks when fitting certain devices to IV traces and stability diagrams respectively, as described above. Therefore, by considering both Supplementary Figure 23 and Supplementary Figure 24, we conclude our quantum approach provides the most robust description of the device B-M dataset.

Supplementary References

1. Simmons, J. G. Generalized formula for the electric tunnel effect between similar electrodes separated by a thin insulating film. *J. Appl. Phys.* **34**, 1793–1803 (1963).
2. Lau, C. S., Mol, J. A., Warner, J. H. & Briggs, G. A. D. Nanoscale control of graphene electrodes. *Phys. Chem. Chem. Phys.* **16**, 20398–20401 (2014).
3. Prins, F. *et al.* Room-temperature gating of molecular junctions using few-layer graphene nanogap electrodes. *Nano Lett.* **11**, 4607–4611 (2011).
4. Limburg, B. *et al.* Anchor groups for graphene-porphyrin single-molecule transistors. *Adv. Funct. Mater.* 1803629 (2018).
5. Frisch, M. J. *et al.* Gaussian 09, Revision A. 02. *Inc., Wallingford, CT* **200** (2009).
6. Sowa, J. K., Mol, J. A., Briggs, G. A. D. & Gauger, E. M. Beyond Marcus theory and the Landauer-Büttiker approach in molecular junctions: A unified framework. *J. Chem. Phys.* **149**, 154112 (2018).
7. Todd, M. D., Nitzan, A., Ratner, M. A. & Hupp, J. T. Electron transfer rates from time-dependent correlation functions. wavepacket dynamics, solvent effects, and applications. *J. Photochem. Photobiol. A* **82**, 87–101 (1994).
8. Nitzan, A. *Chemical dynamics in condensed phases: relaxation, transfer and reactions in condensed molecular systems* (Oxford University Press, 2006).
9. Marcus, R. A. & Sutin, N. Electron transfers in chemistry and biology. *Biochim. Biophys. Acta* **811**, 265–322 (1985).
10. Chidsey, C. E. D. Free energy and temperature dependence of electron transfer at the metal-electrolyte interface. *Science* **251**, 919–922 (1991).
11. Seldenthuis, J. S., van der Zant, H. S. J., Ratner, M. A. & Thijssen, J. M. Vibrational excitations in weakly coupled single-molecule junctions: A computational analysis. *ACS Nano* **2**, 1445–1451 (2008).
12. Sando, G. M. & Spears, K. G. *Ab initio* computation of the duschinsky mixing of vibrations and nonlinear effects. *J. Phys. Chem. A* **105**, 5326–5333 (2001).
13. Lau, C. S. *et al.* Redox-dependent Franck–Condon blockade and avalanche transport in a graphene–fullerene single-molecule transistor. *Nano Lett.* **16**, 170–176 (2015).
14. Bailey, S. *et al.* A study of planar anchor groups for graphene-based single-molecule electronics. *J. Chem. Phys.* **140**, 054708 (2014).
15. Fatayer, S. *et al.* Reorganization energy upon charging a single molecule on an insulator measured by atomic force microscopy. *Nat. Nanotechnol.* **13**, 376 (2018).
16. Scivetti, I. & Persson, M. Frontier molecular orbitals of a single molecule adsorbed on thin insulating films supported by a metal substrate: electron and hole attachment energies. *J. Phys.: Condens. Matter* **29**, 355002 (2017).
17. Limburg, B. *et al.* Charge-state assignment of nanoscale single-electron transistors from their current–voltage characteristics. *Nanoscale* **11**, 14820–14827 (2019).
18. Sowa, J. K., Mol, J. A., Briggs, G. A. D. & Gauger, E. M. Environment-assisted quantum transport through single-molecule junctions. *Phys. Chem. Chem. Phys.* **19**, 29534–29539 (2017).
19. Jortner, J. Temperature dependent activation energy for electron transfer between biological molecules. *J. Chem. Phys.* **64**, 4860–4867 (1976).
